# Bridging the gap: a five stage approach for developing specialty-specific entrustable professional activities

**DOI:** 10.1186/s12909-016-0637-x

**Published:** 2016-04-20

**Authors:** James Kwan, Roslyn Crampton, Lise L. Mogensen, Roslyn Weaver, Cees P. M. van der Vleuten, Wendy C. Y. Hu

**Affiliations:** Medical Education Unit, School of Medicine, Western Sydney University, Sydney, Australia; Westmead Hospital, Sydney, Australia; Graduate School of Health Professions Education, Maastricht University, Maastricht, The Netherlands

**Keywords:** Entrustable professional activities, Assessment design, Qualitative research, Consensus methods, Competence, Emergency medicine

## Abstract

**Background:**

Entrustable Professional Activities (EPAs) are increasingly used as a focus for assessment in graduate medical education (GME). However, a consistent approach to guide EPA design is currently lacking, in particular concerning the actual content (knowledge, skills and attitude required for specific tasks) for EPAs. This paper describes a comprehensive five stage approach, which was used to develop two specialty-specific EPAs in emergency medicine focused on the first year of GME.

**Methods:**

The five stage approach was used to gain consensus on the task, content and entrustment scale for two specialty-specific EPAs in emergency medicine. The participants consisted of twelve clinical supervisors working in the emergency department. The five stages were: 1) Selecting the EPA topic; 2) Developing the EPA content by collecting data from participants using focus group and individual interviews; 3) Drafting the EPAs based on analysis of collected data; 4) Seeking feedback on the draft EPAs from the participants and other stakeholders; 5) Refining and finalising the EPAs based on feedback.

**Results:**

Two specialty-specific EPAs were developed using the five stage approach. The participants reached consensus on the specific tasks and criteria for performance for the two EPAs. They also agreed that both day-to-day (ad hoc) and formal (summative) entrustment decisions were put into practice through the intensity of supervision provided to PGY1 doctors. As a result, a three level entrustment and supervision scale consisting of direct active, indirect active, passive was developed reflecting the shift in the intensity of supervision from close supervision to minimal supervision.

**Conclusions:**

The five stage approach described in this paper was used successfully to develop two specialty-specific EPAs in emergency medicine along with a three level entrustment scale.We propose that the five stage approach is transferable to a range of medical training contexts to design specialty-specific EPAs.

## Background

In the past decade, a re-focusing of medical education has resulted in a conceptual shift from a time-based model towards a competence-based training model [1, 2]. This shift has been recognised worldwide; competence-based educational frameworks have been developed at national and international levels for graduate medical education (GME) training programs. Examples of these frameworks include the Accreditation Council of Graduate Medical Education (ACGME) competencies in the United States [3], the Canadian Medical Educational Directives for Specialists (CanMEDS) [4], Good Medical Practice in the United Kingdom [5] and the Australian Curriculum Framework for Junior Doctors in Australia (ACFJD) [6]. However, there is little agreement on how to meaningfully apply competencies to clinical practice. This has resulted in a tendency to reduce the competencies required to perform complex tasks into simple and potentially trivial behaviours for ease of assessment [7, 8].

Medical school graduates in many countries are required to successfully complete a year of clinical practice under close supervision prior to gaining general registration with the relevant medical board. However, the transition from undergraduate medical education (UME) to GME training is a difficult period for many new graduates, partly due to the lack of a competency framework to promote the vertical integration of competencies across the continuum from UME to GME [9]. For example, doctors in their first year of GME training (PGY1) in Australia are required to complete a rotation in emergency medicine, but there is a lack of practical guidelines to assist graduates to integrate the competencies, which they have learned as students, into what is required for competent emergency medicine practice. This could lead to uncertainty regarding the performance expectations of new graduates, both for supervisors and PGY1s, who usually wish to be well prepared on the first day of their emergency medicine rotation.

Entrustable professional activities (EPAs) have been identified as an approach to bridge the gap between the theoretical aspects of competency-based education and real world clinical practice [2, 10]. The EPA framework was conceptualised by ten Cate [2] as a way to operationalise the assessment of competencies, formulating them as the essential professional activities that a competent doctor can be entrusted to perform. EPAs have been described as “units of professional practice, defined as tasks or responsibilities to be entrusted to a trainee once sufficient specific competence is reached to allow for unsupervised practice. They are independently executable within a time frame and observable and measurable in the process and outcome” and therefore can be used to make entrustment decisions [11].

EPAs can be used by supervisors to contextualise abstract competencies in real world practice [10], to meaningfully assess the progress and capabilities of new graduates, and provide clearer expectations for students regarding the clinical tasks they will be required to perform as graduates. Further, EPAs also offer curriculum developers and teachers tangible goals for aligning teaching and training with clinical practice [12], across the continuum from UME to GME [9]. This has been found to be relevant in diverse medical training systems, from the Netherlands [11, 13, 14], US [15–19], Australia, New Zealand [20, 21] and Singapore [22].

The Association of American Medical Colleges has published 13 core EPAs which medical graduates are expected to be entrusted with on the first day of GME training [19], although these address generic skills rather than expectations for specific specialties required at graduation[9]. While specialty-specific EPAs for GME have been developed in specialties such as psychiatry [20, 21], paediatrics [7, 11], obstetrics and gynaecology [10], internal medicine [15, 16, 18], gastroenterology [23], family medicine [17], and surgery [22], in some cases they may be set at a competency level which is too advanced for new graduates.

Furthermore, the EPA development process requires further work, particularly with regard to an accepted approach to guide EPA design. Currently, many papers are conceptual, such as viewpoints or commentaries, with little empirical research [2, 10–12, 21, 22, 24–27]. Several papers directly focus on developing EPAs [15–20] or particular aspects directly related to developing EPAs [27], or were using EPAs as part of their research process in developing competence-based curricula [14, 28, 29]. Where methods were described, the focus was typically on the first step of EPA development: identifying suitable clinical tasks for EPAs. These methods often involved a modified Delphi technique or similar expert-panel approaches [15, 17, 18, 20]. What is missing is a rigorous methodology for the second step: producing the actual content for the different sections of EPAs, such as the required knowledge, skills and attitudes for particular tasks. Although general guidance for designing the content of EPAs has been provided in previous literature [13, 30], descriptions of the process lack sufficient detail for effective and consistent implementation, particularly with regard to determining content sources. For example, guidelines on defining the required knowledge, skills and attitudes suggest referring to manuals, books, protocols or instruction booklets [13, 30] but most reports do not explain who chooses this content, with one report specifying tutors [21]. Developing an explicit and comprehensive approach will provide practitioners and educators with clearer steps for developing EPAs irrespective of the training context, thereby reducing the time and effort spent in determining a development process for each occasion.

In order to address the research gap in methodology and the lack of a consistent approach for producing the actual content for EPAs, we describe a comprehensive five stage approach based on qualitative methodology, which was used to develop two specialty-specific EPAs in emergency medicine focused on PGY1 to be performed with minimal supervision prior to transitioning to the second year of GME (PGY2). In contrast to previous studies, which have used the Delphi or nominal group technique to simply identify suitable tasks rather than the actual content for proposed EPAs [15, 17, 20], our method involves the use of in-depth focus group and individual interviews with clinical supervisors working in the emergency department, to gain consensus not only on the task but also the detailed content for the proposed EPAs.

## Methods

A qualitative study design involving focus groups and individual interviews was chosen to collect and analyse the opinions of clinical supervisors to gain consensus on the EPA task, detailed content and an entrustment scale for two specialty-specific EPAs in emergency medicine. The study was approved by the University of Western Sydney Human Research Ethics Committee (Reference: 12/027745/H9989). Written informed consent was sought and obtained from all participants and procedures for the responsible conduct of research followed, including use only of de-identified raw transcript data in the results section of this paper, to ensure the anonymity and confidentiality of the participants’ responses.

### Study setting and participants

This study was conducted in Australia where medical graduates are required to successfully complete one year of clinical practice under close supervision prior to receiving general registration with the Medical Board of Australia and progressing onto generalist or specialist GME training in PGY2. During this year of supervised clinical practice, all doctors must complete a rotation in emergency medicine. In order to address the lack of suitable EPAs during this important period of supervised training, PGY1 was chosen as the focus for the development of two specialty-specific EPAs in emergency medicine to demonstrate the utility of the proposed five stage approach for developing EPAs.

Participants were doctors from a large urban hospital in Sydney, Australia. The hospital is the centre of one of the largest postgraduate training networks in the state of New South Wales, training 114 PGY1 doctors per year in 31 specialties and subspecialties. Purposive sampling was used to select emergency medicine physicians with different levels of experience in supervision of PGY1 doctors, who were actively supervising PGY1 doctors and would therefore be “information-rich” [31] participants for the research.

### A five stage approach for developing EPAs

The approach incorporated five defined stages (Table 1), based on the theoretical framework described by ten Cate for designing EPAs [11].

**Table 1:** Five Stage approach for developing specialty-specific EPAs using qualitative research methods

1. Select the EPA topic based on tasks that cover essential work in a specific environment. Ensure that they are observable and measurable, and include knowledge, skills and attitudes that reflect one or more competencies	
2. Develop the EPA content by collecting qualitative focus group and/or? individual interview data from participants	
3. Draft the EPAs based on thematic analysis of collected data to populate domains in accepted EPA formats	
4. Seek feedback on the draft EPAs from participants and other stakeholders as a form of member checking	
5. Refine and finalise the EPAs based on feedback	

### Stage 1: selecting the EPA topic

EPAs generally follow a format that includes a title, justification, description, link to a relevant competency framework, the knowledge, skills, and attitudes required to undertake the task, sources of information to assess progress and the basis for formal entrustment decisions [11, 13, 22]. Recommendations about EPA design in the literature [2, 10] suggest that it is important that the tasks cover essential work in a specific environment, that they are observable and measurable and include knowledge, skills and attitudes that reflect one or more competencies. The two tasks selected for EPA development were among the most common presentations in emergency medicine [32–34]: (1) an adult presenting with acute chest pain and (2) an elderly patient presenting after a fall .

### Stage 2: developing the EPA content by collecting data from participants using focus group and individual interviews

To develop EPA content, data was collected via focus groups and telephone individual interviews from participants consisting of emergency medicine physicians with experience in supervising PGY1s. Three focus groups were formed according to professional background and years of experience consisting of: three early stage advanced trainees (postgraduate year four and above), two late stage advanced trainees and one career medical officer (postgraduate years six and above), and four Specialists. The participants were interviewed in small groups separated by their current position. This separation was made to avoid potential hierarchy effects that may potentially constrain the response of participants with different levels of authority and power, particularly where participants have supervisor-supervisee relationships [35]. The focus group interviews lasting approximately one hour were facilitated by three research team members (LLM, RW, WCYH) with experience in focus group facilitation but no professional connection with participants.

In the focus groups, participants were presented with two patient scenarios on the chosen topics (1) an adult presenting with acute chest pain and (2) an elderly patient presenting after a fall. Semi-structured questions about the capacities of new PGY1s and supervision strategies, related to these presentations were asked (Table 2). These questions were based on the recommendations for EPA design, to define content for each section of the EPA.

**Table 2:** Focus group and individual interview questions

1. What skills would a new intern need to perform this task safely and competently?	
2. How would you supervise a new intern performing this task?	
3. What would you look for to decide how much supervision a new intern needs to perform this task?	
4. Under what circumstances would you need to supervise an intern more closely?	
5. When would you trust a new intern to perform this task on their own? 6.When a new intern is having difficulty putting a case together, what do you do to help them develop this skill?	
7. How would you assess how well a new intern is performing this task?	
8. How would your assessment differ or change for different times in the rotation? For example at the beginning, versus mid term versus end of term?	
9. Is there anything else you would like to add about teaching and assessing new interns in tasks that require them to assess, synthesise and prioritise patient presentations?	

In addition to the focus group interviews, three telephone interviews of 30 to 60 minutes duration were conducted after the workshop by two of the researchers (RW, LLM). Two interviews were conducted to include participants unable to attend the workshop, using the same patient scenarios and semi-structured questions as in the focus groups. One interview was conducted with a participant to seek clarification on this participant’s responses during the focus group interviews. Our intent was to gather a broad sample of views from relevant stakeholders and for reasons of feasibility and value both focus groups and individual interviews were used. While we acknowledge that in qualitative research the two data collection methods have different purposes (e.g. elicitation of group consensus views and interpersonal dynamics versus individual viewpoints), for the purposes of our study either are effective as a practical approach to eliciting the views of relevant people.

All workshop and telephone interviews were audio recorded and transcribed verbatim by a third party. The transcriptions were checked for accuracy against the audio files and de-identified for participant privacy by two members of the research team (LLM, RW).

### Stage 3: drafting the EPAs based on analysis of collected data

To structure the analysis, each researcher was provided with a pre-determined MS Word template of four questions closely aligned with the semi-structured interview questions related to EPA content, entrustment and clinical supervision. The questions were:

1. What knowledge, skills and attitudes are required to integrate clinical findings to perform this EPA at the level of a PGY1?
2. What cues and observations does the respondent use to assess a PGY1 performing this activity?
3. What evidence does the respondent need to decide whether to ‘trust’ the PGY1 to perform this activity independently?
4. What strategies does the respondent use in teaching the PGY1 to effectively perform this task?

Each transcript then underwent independent analysis by both an emergency medicine content expert and a non-content expert educational researcher on the research team to identify a priori ideas as expressed by the participants. These analyses were then collated and compared. Differences between analyses were discussed and where appropriate, divergent themes were identified, and any conflicting interpretations resolved through iterative discussion and refinement of the identified ideas and themes. Content experts and non-content experts bring different perspectives that may influence data analysis [36] and adopting multiple perspectives enhances the depth and rigour of the analysis, as well as triangulation [37]. Although the template guided analysis for the purpose of developing the EPAs, analysts were not limited to the framework if additional themes were identified. Data saturation was evident when no new themes emerged from the individual interviews conducted after the workshop.

Following this process, research team members (LLM and RW) integrated the emerging themes using Microsoft Excel^©^ by reviewing themes across each participant group. Common themes across participant groups were selected to populate each section of the first draft EPAs.

### Stage 4: seeking feedback on the draft EPAs from the participants and other stakeholders

To ensure the EPAs adequately reflected the participants’ insights, written feedback on the draft EPAs was sought from participants, with a specific request to provide feedback on the extent to which the EPAs reflected their views, and on the clarity and usefulness of the EPAs for teaching and assessing PGY1s in emergency medicine, as a form of member checking. Additionally, PGY1 doctors and undergraduate medical students were invited to provide feedback from the learner’s perspective.

### Stage 5: refining and finalising the EPAs based on feedback

Feedback data on the draft EPAs from participants, PGY1s and students was collated and incorporated into the two final specialty-specific EPAs in emergency medicine.

## Results

Twelve clinical supervisors were engaged in the five stage approach as participants to develop EPA content (Table 3). Of these, 11 contributed up to the final stage of EPA development. Feedback on the draft EPA was also received from two postgraduate doctors and one final-year undergraduate medical student.

**Table 3:** Participant demographics

Characteristics	Participants, *N*?=?12
Gender	
Female	6
Male	6
Age	
30–39	4
40–49	6
50+	2
Language	
English	12
Other languages	4
Experience since graduation	
5+ years	2
10+ years	2
15+ years	4
25+ years	4
Other qualifications	
Postgraduate (Master level degree)	2
Professional (Fellowship/Membership of Professional Colleges)	6
Current position	
Early Advanced trainee	3
Late Advanced trainee	2
Career medical officer	1
Early-mid career Specialist	3
Senior Specialist	3
Supervision experience – postgraduate doctors	
<5 years	2
5+ years	4
10+ years	3
15+ years	2
20+ years	1

The following section presents our findings from the process of developing EPAs in the two chosen key tasks for PGY1 doctors in the ED (Tables 4 and 5): ‘building knowledge, skills and attitudes’ (KSA), and ‘assessment process and methods’, and ‘basis of entrustment’. De-identified quotes from each participant group have been used to enhance the presentation of the results.

### Designing the EPAs

#### Building knowledge, skills and attitudes

The key content provided by the participants for the domain focusing on KSAs was sufficiently complex to support the development of higher level synthesis and reasoning skills. To ensure the KSAs were applicable to the local context, they were aligned with the Australian Curriculum Framework for Junior Doctors [6]. However, in line with recommendations for EPA design [11], KSAs can be modified as required to suit the context, specific needs and relevant competency frameworks of emergency departments in other settings and locations.

**Table 4:** EPA – Managing adult patients with acute chest pain

Title	Acute chest pain
Justification	Chest pain is one of the most common presentations in the emergency department. The ability to conduct an accurate initial assessment of acute chest pain in adult patients is important in order to manage potentially life-threatening conditions.
	In the emergency department, initial assessment of patients with acute chest pain requires the integration of multiple competencies and the ability to execute these in a busy clinical environment with multiple distractions.
Description	PGY1s must be able to assess, synthesise and prioritise key steps required in managing the care of an adult patient presenting with acute chest pain
	They must have the ability to conduct a patient history and examination relevant to acute chest pain in a timely manner They must select, justify and interpret appropriate investigations and synthesise findings to formulate a working diagnosis. Interns must be able to commence initial therapeutic steps within guidelines applicable to the setting in which they work
	They must have knowledge of causes, investigations and treatment options relevant to acute chest pain, and be able to recognise typical presentations of life-threatening diseases
	PGY1s must also have the ability to recognise the signs and symptoms of a critically ill patient, and seek supervisory assistance with appropriate urgency
Link with ACFJD competencies	Clinical management – Safe patient care (Systems; Radiation safety); Patient assessment (History and examination; Problem formulation; Investigations; Referral and consultation); Emergencies (Assessment; Prioritisation); Patient management (Management options)
	Communication – Patient interaction (Respect); Managing information (Written); Working in teams (Team structure; Case presentation)
	Professionalism – Doctor and society (Professional standards); Professional behaviour (Professional responsibility; Time management; Personal well-being)
	Skills and procedures – General (Measurement; Interpretation of results; Intravenous; Diagnostic); Cardiopulmonary
	Clinical problems and conditions – Circulatory
Required knowledge, skills and attitudes	Knowledge
	Demonstrates knowledge of chest pain causes
	Demonstrates knowledge of signs and symptoms indicating patient is critically ill
	Demonstrates knowledge of differential diagnoses related to chest pain, such as aortic dissection, pulmonary embolism, and myocardial infarction
	Demonstrates knowledge of relevant investigations and treatment options for chest pain
	Demonstrates knowledge of local guidelines in managing patients with chest pain (such as chest pain pathways)
	Skills
	Recognises signs of critical illness and can ask for help when needed with appropriate urgency
	Takes a focused, relevant and succinct patient history in a timely manner
	Performs a relevant and focused physical examination, including vital signs
	Synthesises information to formulate provisional diagnosis
	Performs procedural skills (venepuncture, cannulation)
	Selects, requests and can justify relevant investigations (ECG, chest x-ray, blood tests)
	Interprets relevant investigations (ECG, chest x-ray, blood tests)
	Recognises abnormal results from investigations (ECG, chest x-ray, blood tests)
	Simple pain management within appropriate guidelines for the setting
	Formulates and can justify initial management plan
	Maintains accurate and thorough documentation
	Presents case clearly and succinctly to senior doctors and other staff
	Attitudes
	Adheres to professional standards
	Aware of own limitations and asks for help appropriately
	Respects patient privacy and confidentiality
	Treats patients courteously and respectfully
	Respects other health professional team members
	Behaves in ways to mitigate the personal health risks of emergency medicine, such as fatigue and stress
Sources of information to assess progress	This EPA is continuously assessed during clinical supervision of PGY1s using direct observation, structured interviewing, case presentation and multi-source feedback.
Entrustment and supervision scale	Supervision of PGY1s is required with the supervisor present in the emergency department. However, the intensity of supervision varies according to the individual PGY1’s ability to perform the EPA. The 3 levels of decreasing intensity of supervision reflect the levels of entrustment.
	Level 1: Direct active – Full supervision at bedside. After the supervisor’s initial assessment of the patient, the PGY1 assesses the patient with regular prompting and feedback from the supervisor.
	Level 2: Indirect active – Partial supervision within line of sight. Supervisor pre-prompts PGY1 to assess the patient. The PGY1 reports back his or her assessment of the patient to the supervisor.
	Level 3: Passive – Full entrustment with the supervisor present in the emergency department. The supervisor entrusts the PGY1 to initiate assessment of the patient and report back his or her findings with minimal prompting and feedback.
Estimated stage of training when level 3 (Passive) is to be reached	End of the emergency medicine rotation in the first year of GME training (PGY1)
Basis for formal entrustment decisions	The following activity will be entrusted at level 3 when the supervisor is confident that the PGY1 has the knowledge, skills and attitudes to perform the activity at an acceptable standard and that the intern knows when to ask for help in a timely manner.

**Table 5:** EPA – Managing elderly patients following a fall

Title	Fall in the elderly
Justification	Fall injuries are a common and potentially complex presentation in the emergency department. Appropriate management may be particularly critical with elderly patients. Therefore, the ability to conduct an accurate initial assessment of an elderly patient admitted following a fall is important in order to manage patients with potentially complex conditions.
	In the emergency department, initial assessment of elderly patients admitted following a fall requires the integration of multiple competencies and the ability to execute these in a busy clinical environment with multiple distractions.
Description	PGY1s must be able to assess, synthesise and prioritise key steps required in managing care of an elderly patient (aged 65 and older) presenting after a fall.
	They must have the ability to conduct a patient medical and social history in a timely manner to establish the cause/s of fall, the injuries sustained in fall and the functional and social implications of the fall. They must undertake an appropriate trauma examination, and be able to select, justify and interpret appropriate investigations, and synthesise findings to formulate a working diagnosis. PGY1s must be able to commence initial therapeutic steps within guidelines applicable to the setting where they work.
	They must have knowledge of trauma investigations and treatment options relevant to falls generally and to those specific to caring for elderly patients. Their knowledge must incorporate physiology in the context of elderly patients.
	Interns must also have the ability to recognise the signs and symptoms of a critically ill patient, and seek supervisory assistance with appropriate urgency.
Link with ACFJD competencies	Clinical management – Safe patient care (Systems); Patient assessment (History and examination; Problem formulation; Investigations; Referral and consultation); Emergencies (Assessment; Prioritisation); Patient management (Management options; Therapeutics; Pain management; Discharge planning)
	Communication – Patient interaction (Context; Respect; Meetings with families or carers); Working in teams (Team structure; Case presentation)
	Professionalism – Doctor and society (Access to health care; Culture, society and health care; Professional standards); Professional behaviour (Professional responsibility; Time management; Personal well-being)
	Skills and procedures – General (Measurement; Interpretation of results); Trauma
	Clinical problems and conditions – Neurological; Critical care/Emergency
Required knowledge, skills and attitudes	Knowledge
	Demonstrates knowledge of trauma symptoms and management procedures
	Demonstrates knowledge of effects of trauma in the elderly patient
	Demonstrates knowledge of normal functioning, vital signs, and hemodynamic responses in the elderly patient
	Demonstrates knowledge of reasons for relevant investigations and treatment options for elderly patients admitted following a fall
	Demonstrates knowledge of interactions between trauma, co-morbidities and pre-morbid conditions
	Skills
	Recognises signs of critical illness and can ask for help when needed with appropriate urgency
	Takes a focused, relevant and succinct patient history (medical and social) in a timely manner
	Ascertains cause/s of fall
	Performs a relevant and focused trauma assessment
	Synthesises information to formulate provisional diagnosis
	Performs basic procedural skills (for example, suturing)
	Selects, requests and can justify relevant investigations (CT, x-ray)
	Interprets relevant investigations (x-ray)
	Recognises abnormal results from investigations
	Simple pain management within appropriate guidelines for the setting
	Formulates and can justify initial management plan, within the context of the patient’s unique social circumstances and co-morbidities/pre-morbid conditions
	Presents case clearly and succinctly to senior doctors and other staff
	Attitudes
	Adheres to professional standards
	Aware of own limitations and asks for help appropriately
	Respects patient privacy and confidentiality
	Treats patients and patients’ family members courteously and respectfully
	Respects other health professional team members
	Behaves in ways to mitigate the personal health risks of emergency medicine, such as fatigue and stress
Sources of information to assess progress	This EPA is continuously assessed during clinical supervision of PGY1s using direct observation, structured interviewing, case presentation, and multi-source feedback.
Entrustment and supervision scale	Supervision of PGY1s is required with the supervisor present in the emergency department. However, the intensity of supervision varies according to the individual PGY1’s ability to perform the EPA. The 3 levels of decreasing intensity of supervision reflect the levels of entrustment.
	Level 1: Direct active – Full supervision at bedside. After the supervisor’s initial assessment of the patient, the PGY1 assesses the patient with regular prompting and feedback from the supervisor.
	Level 2: Indirect active – Partial supervision within line of sight. Supervisor pre-prompts PGY1 to assess the patient. The PGY1 reports back his or her assessment of the patient to the supervisor.
	Level 3: Passive – Full entrustment with the supervisor present in the emergency department. The supervisor entrusts the PGY1 to initiate assessment of the patient and report back his or her findings with minimal prompting and feedback.
Estimated stage of training when level 3 (Passive) is to be reached	End of the emergency medicine rotation in the first year of GME training
Basis for formal entrustment decisions	The following activity will be entrusted at Level 3 when the supervisor is confident that the PGY1 has the knowledge skills and attitudes to perform the activity at an acceptable standard and that the intern knows when to ask for help in a timely manner.

Participant feedback confirmed that the KSAs could reasonably be expected of PGY1 doctors at the end of the rotation, suggesting only minor adjustments to the KSAs to improve the clarity or specificity of the wording. For example, the draft wording in skills for the acute chest pain EPA “recognizes abnormal results of investigations” was changed to specify ECG, chest x-ray, and blood tests. Students’ and PGY1 doctors’ feedback did not suggest any changes; they agreed that this section was the most useful part of the EPA because “it explicitly states what we need to be able to perform and know”.

#### Assessment process and methods

There was consensus that assessment of PGY1 doctors’ performance against the EPA domains should be an ongoing process involving observation, direct questioning and case presentations during ward rounds and handovers and global feedback from other supervisors. The participants also noted that they would double check the accuracy of the case presentations against their own assessment of the patient and their notes made in the patient records. Interestingly, there was no mention of specific timing of assessments but that the assessment of the PGY1 doctor’s ability and behaviour occurred continuously.

In keeping with the participants’ accounts of supervision ‘on the run’, the sources of information to assess progress section in the EPA have only been briefly described in non-prescriptive terms, to allow clinical supervisors the flexibility to opportunistically choose the most appropriate KSA and method at the time of assessment.

Feedback from participants suggested that the section on sources of information to assess progress reflected clinical practice and no changes were suggested.

#### Basis of entrustment: expectations of new PGY1s

A near universal theme was the low expectations the participants had of new PGY1 doctors. This influenced the design of the EPA levels of entrustment. Most participants were either unsure or had very minimal expectations of the abilities of new graduates at the beginning of the ED rotation, regardless of the medical program the PGY1 doctor had graduated from. Because of this uncertainty, most stated that they would commence supervision assuming that PGY1 doctors had very limited knowledge and skills. As one participant described it, “my expectation of an intern is at the lowest level possible and I mean that in a complimentary way, like they’re always very enthusiastic [… but] certainly for me I think most interns, day one, the skill base is not achieved at that time” (Specialists).

These low expectations were linked to difficulties in the transition from student to PGY1 doctor as well as the ED environment itself. It was noted that this transition can be a difficult process, and PGY1 doctors were often unprepared for the demands of clinical practice: “Although medical school is good, it’s still really not prepared them in some ways for being at the coalface” (Specialists). When PGY1 doctors are “thrown into the thick of it” they must quickly adapt to the increased responsibility of being a doctor, and “stop being students” (Specialists).

EPAs need to be very specific to the clinical context where they are to be used. In our patient scenarios, the unique nature of the ED environment, with its urgent, often life-threatening cases, was a further reason why entrustment levels were so important to consider. Participants commented that PGY1 doctors experience stress in a “chaotic”, “scary” setting. One described this early experience as being “launched into the arena like gladiators” (Early advanced trainees). Unlike other clinical rotations, ED requires PGY1 doctors to be “real doctors” not “secretaries” (Advanced trainees; Specialists). The stress of this environment meant that PGY1 doctors often floundered and lacked confidence, which affected their skills and general competence. Participants found that they were having to build confidence in PGY1 doctors to enable them to perform successfully in the ED environment, thus began supervision without high expectations of PGY1 doctors’ abilities.

A further consideration for the basis of entrustment was the common observation that most PGY1 doctors struggle to synthesise information and produce a confident diagnosis. Whilst there was agreement among the participants that most PGY1 doctors have the knowledge and skills required for assessment and physical examination of patients, supervisors often had to prompt and guide PGY1 doctors through the next steps in order to reach a diagnosis and articulate a treatment plan: “contextualising what disease process is happening and diagnosis … when they first come, they really struggle with that” (Advanced trainees).

It was acknowledged that the ability to synthesise information takes time to develop. For some PGY1 doctors this process is slower than for others. “I’ve seen some doctors who take three years to achieve that [… but] I’ve got some interns now that seem to be on track within a couple of weeks” (Specialists).

#### Basis of entrustment: factors influencing entrustment decisions

Uncertainty about the abilities of new PGY1 doctors influenced participants’ decisions to entrust them with clinical duties and there was strong agreement on the basis on which PGY1 doctors are awarded entrustment. Three key factors affected the participants’ day-to-day or ad hoc entrustment decisions: familiarity, patient condition and ED environment. Firstly, they needed to be familiar with the PGY1 doctor’s level of performance, through the PGY1 doctor consistently and reliably demonstrating KSAs in patient assessment, examination and investigation. Once confident that the PGY1 doctor was able to integrate KSAs and synthesize findings, the participants said that they would gradually trust PGY1 doctors with more tasks and less close supervision. Secondly, entrustment depended on the patient’s condition; if unstable or critically ill, close supervision or taking control of patient management was warranted, regardless of their familiarity with the PGY1 doctor. “It’s the red flags in the triage that made you think, I can’t let that one go” (Advanced trainees). Finally, ad hoc entrustment decisions were influenced by the state of the ED environment at the time. For example, if the emergency department were particularly busy, the participants said that PGY1 doctors would sometimes be required to undertake tasks with little supervision due to clinical service demands placed on their supervisors. “It’s often dependent on the situation in the department in terms of patient flow and time pressures … ignoring that factor would be un-sensible [sic], because it does definitely affect the way that you supervise” (Advanced trainees).

#### Basis of entrustment: entrustment and supervision scale

These findings informed the entrustment and supervision scale section of the EPAs. Regarding the three factors affecting ad hoc entrustment, the participants noted the need to draw upon a range of supervision methods and supervise at varying levels of intensity. Acknowledging the mandatory requirements for supervision of all PGY1 doctors, analysis revealed three formal or summative levels of entrustment and supervision:

- Level 1: Direct active
  This was described by participants as direct bedside supervision where the PGY1 doctor and their supervisor would assess the patient together. Most explained this as their initial approach to supervision with PGY1 doctors. Some described this as “hovering around the patient” or keeping a “short leash” (Specialists). Others framed this as a “collaborative approach” (Specialists), where supervisor and the PGY1 doctor would be “working in tandem” (Early advanced trainees), to conduct the patient assessment and make decisions about investigations and treatment.
- Level 2: Indirect active
  At this level, participants allowed PGY1 doctors to see patients unaccompanied but only after directing them first with questions to ask and issues to consider in their patient assessment. In the words of one participant, “I guess the most common thing is I often plant a seed in their head before they go in […] I will say, ‘Let’s see what injuries the patient has got from the fall, but we need to find out why she fell’ or ‘Make sure you find out, you know, is she on any anticoagulants’, things that would be raising further alarm bells of potential catastrophic injuries or outcomes” (Specialists).
- Level 3: Passive
  This level involved allowing PGY1 doctors to assess the patient independently and report back as needed: “we normally just send them to see the patients and they come and report back to us, and then we go and review the patient” (Specialists). The participants suggested that EPA is entrusted at Level 3 when the supervisor is confident that the PGY1 doctor has the knowledge skills and attitudes to perform the activity at an acceptable standard and that the PGY1 doctor knows when to ask for help in a timely manner. We anticipate that level 3 (passive) entrustment will be reached by the end of the emergency medicine rotation in PGY1.

During member checking, 10 of 12 participants agreed that: a) these three levels accurately reflect their views on entrustment and supervision, b) the levels are appropriate for PGY1 doctors, and c) the entrustment model provides a suitable guide to adapt supervision according to the ability of individual PGY1s. Two suggested that the first level was more appropriate for students than for postgraduate doctors. Feedback from the student and PGY1s agreed that the entrustment model was “a useful part of the EPA” that “defines the level of supervision well”.

## Discussion

We describe a comprehensive five stage approach using in depth focus groups and individual interviews, to gain consensus on the task, content and entrustment scale of two specialty-specific EPAs as a focus for assessment for new medical graduates in emergency medicine during PGY1.

Traditionally, Delphi or nominal group techniques have been used to identify tasks for proposed EPAs [15, 17, 20]. Our study describes a generic approach to EPA development using in depth focus group and individual interviews to gain consensus on the task, content and entrustment scale applicable to range of training contexts. In contrast to the nominal group technique where hierarchical relationships may exist between participants, homogeneity within our focus groups avoided potential hierarchical supervisor-supervisee effects that may limit the responses of some of our participants. Variability in our participants’ opinions has been addressed by independent analysis of the focus group and individual interviews to gain consensus on both the task and the detailed content of each section of the EPA. In addition, care has been taken to engage as participants clinical supervisors who are experienced in supervising the targeted level of learners in that particular setting, in order to maximise the validity of the EPAs being developed.

In addition, the use of intensive interviews has enabled the identification of an unexpected finding: the very low expectations by participants of PGY1 doctors commencing ED rotations. It appears that the experience of supervisors in particular contexts may diverge from the expectation that graduates have reached a certain level of competency, as defined by the generic national graduate outcomes specified by the Australian Medical Council, the accrediting body for all medical programs in Australia [38]. This apparent inconsistency may in part be due to the different competency expectations for specific specialties between individual medical schools [9] and highlights the importance of considering specific settings and contexts when designing EPAs.

We suggest that our findings of low expectations of PGY1 doctors perhaps relate to the unique environment of the ED with a high proportion of urgent and often life threatening cases and uncertainty about the abilities of PGY1 doctors. This finding not only has implications for EPA development, but for undergraduate medical programs to carefully consider the specific competencies required for settings in which new graduates may practice. Perhaps through a process of identifying EPAs as a focus for assessment, undergraduate curriculum designers can better ensure that students are prepared for graduate practice, and supervisors can be more confident of the foundational capabilities of new graduates in their particular setting, and thus more efficiently allocate an appropriate level of entrustment and supervision.

Whilst the basis on which supervisors make entrustment decisions is complex, our findings suggest that familiarity with the PGY1 doctor, patient factors and the ED environment all affect ad hoc entrustment decisions, and these are consistent with the factors known to affect supervisors’ ad hoc entrustment decisions; the relationship between supervisor and trainee, context and task [39–41]. These ad hoc entrustment decisions were assessed through cross checking of the supervisor’s clinical findings against that of the PGY1 doctor’s, which is consistent with findings by Kennedy and her colleagues in an emergency medicine setting [42]. In our study, entrustment decisions appeared to be put into practice through the intensity of supervision provided by our participants which supports an entrustment scale incorporating the intensity of supervision as a construct as proposed by Chen and her colleagues [9].

Our method has also highlighted another unexpected finding; that ad hoc entrustment *increased* when the ED was under pressure due to patient care demands. This is counterintuitive as it could be assumed that there is a greater need for supervision when patient acuity and time pressure is increased. This phenomenon has been previously observed in the context of under-resourced clinical environments in the United States. One study showed that trainees may be given more independence, for example during night shifts, despite not having been previously afforded this level of trust by their supervisors [43]. From the learner’s perspective, our study suggests that using EPAs to provide a higher level of autonomy earlier in the rotation may allow supervisors to more quickly judge a PGY1’s trustworthiness. From a professional and organisational perspective, the timely assessment and management of patient care may take precedence over supervisory precautions, which might be undertaken at different times and under different circumstances. It is likely that this shift in entrustment is not infinite; there will be a threshold under which direct supervision will be required. By engaging actual supervisors in the process of determining an EPA and articulating the levels of summative entrustment and supervision in clinical practice, the location of this threshold can be much better understood by supervisors, PGY1 doctors and graduating students.

Based on our findings related to the factors affecting ad hoc entrustment and the intensity of the supervision provided by our participants to PGY1 doctors in the ED, we propose three levels of summative entrustment related to the intensity of supervision highly relevant to this context: active, indirect active and passive. These three can be mapped between levels 2 (practice EPA under direct, proactive full supervision) and 3 (practice EPA under reactive/on-demand supervision) of the five levels of supervision under the current GME entrustment and supervision scale described by ten Cate [30, 44]. Our findings support the proposal by Chen and her colleagues to expand the lower ends of the entrustment scale to allow for a more granular progression of autonomy during the earlier stages of training [9]. Acknowledging the mandatory requirement in our setting that all PGY1 doctors are to be supervised at all times in the ED, it may not be practical for level 4 (practice EPA unsupervised) to be reached by the end of PGY1.

### Limitations

The findings in this study are limited to emergency medicine in the urban hospital setting. Whilst some of the EPA content may be transferable to similar settings, we argue that the main value of our research is to comprehensively describe a generic five stage approach for developing EPAs that can potentially be applied in a broad range of settings. It may also be argued that not all settings will have access to a research team of content and methodological experts to develop an EPA. However, we believe that our approach is suitable for non-methodology expert users; in other contexts, members of a multidisciplinary team from different health professional backgrounds, or staff members from other departments in the health service may provide the alternative perspectives required for triangulation of the findings.

Our findings have also been useful for identifying what, and how, further work could be done to develop a complete set of EPAs for medical graduates at the transition to supervised clinical practice in emergency medicine. The development of EPAs through a process that engages with, and results in a focus on assessment which is meaningful to supervisors, should increase the confidence of supervisors to make entrustment decisions of PGY1 doctors. Areas for further exploration and methodological development include the crucial interaction between patient, trainee, supervisor, environment and task-related factors. Using qualitative research approaches, such as ethnography, may lead to greater insights on how the interplay between these factors affects individual supervisors’ summative entrustment decisions.

## Conclusions

This study details a comprehensive five stage approach using focus groups and individual interviews to gain consensus on the detailed content for two specialty-specific EPAs in emergency medicine and a context relevant entrustment and supervision scale for PGY1. The proposed five stage approach provides a generic approach for EPA design which can be transferred to a range of training contexts.

### Availability of data and materials

Ethical approval has not been sought for the publication of de-identified transcripts of focus group and individual interviews in this study.

## Abbreviations

ACGME: Accreditation Council of Graduate Medical Education
ACJFD: Australian Curriculum Framework for Junior Doctors in Australia
CanMEDS: Canadian Medical Educational Directives for Specialists
EPA: Entrustable Professional Activity
PGY1: Postgraduate Year 1 (first year of GME training)
